# On the Maximum Obtainable Purity and Resultant Maximum Useful Membrane Selectivity of a Membrane Separator

**DOI:** 10.3390/membranes14060143

**Published:** 2024-06-19

**Authors:** Sean-Thomas B. Lundin, Ayumi Ikeda, Yasuhisa Hasegawa

**Affiliations:** Research Institute of Chemical Process Technology, National Institute of Advanced Industrial Science and Technology (AIST), 4-2-1 Nigatake, Miyagino-ku, Sendai 983-8551, Japan; a-ikeda@aist.go.jp (A.I.); yasuhisa-hasegawa@aist.go.jp (Y.H.)

**Keywords:** membrane separator modeling, process optimization, carbon capture and utilization (CCUS), CO_2_ separation, binary gas separation

## Abstract

Design considerations concerning the maximum purity of a membrane separator, and the resultant maximum effective selectivity of the membranes were explored by modeling a binary gas membrane separator (pressure-driven permeance) using a dimensionless form. Although the maximum purity has an analytical solution at the limit of zero recovery or stage cut, this solution over-predicts the obtained purity as the recovery is increased. Furthermore, at combinations of high recovery, low feed mole fraction, and low pressure ratio, the maximum purity becomes independent of selectivity above some critical selectivity. As a consequence of this purity limitation, a maximum selectivity is defined at which further increases in selectivity will result in less than a 1% change in the final purity. An equation is obtained that specifies the region in which a limiting purity is less than unity (indicating the existence of a limiting selectivity); operating at less than the limiting pressure ratio results in a purity limitation less than unity. This regime becomes larger and more significant as the inlet mole fraction decreases (e.g., inlet feed mole fraction of 10% and pressure ratio of 100 results in a maximum useful membrane selectivity of only 130 at 95% recovery). These results suggest that membrane research should focus on increasing permeance rather than selectivity for low-concentration separations. The results found herein can be used to set benchmarks for membrane development in various gas separation applications.

## 1. Introduction

Gas separations represent a growing market segment, with several potential applications including carbon capture [1,2], hydrogen purification [3], and chemical upgrading [4]. Compared to pressure-swing adsorption or cryogenic distillation, membrane separators do not require the energy-intensive processes of pressure or thermal cycling. However, historically, membranes have suffered from low selectivity or low permeance that has prevented them from being economically viable for the large number of potential applications available [5]. Yet, as membrane technologies improve, so do the economics [6]. This has resulted in an increased interest in membrane separations in the research community, with a wide range of target separations and numerous techno-economic assessments being published.

Potential gas separations span a wide range, including the most common applications of hydrogen generation, air purification, and CO_2_ capture. With these applications, a range of potential feed gas compositions is possible, with concentrations as high as 75% for hydrogen production from ammonia [7], to as low as ca. 400 ppm for direct air capture of CO_2_ [8]. Additionally, a large number of potential separations sit within a moderate concentration range, including O_2_ purification from air (20%) [9], and CO_2_ separation from industrial flue gases (typically 5–30%) [10,11]. Thus, any modeling study analyzing the separation of membranes from a general perspective must span a wide range of inlet concentrations. Although most studies focus on a small number of applications and thus limit the feed conditions to within a limited range.

The primary challenges in modeling membrane separators include the presence of concentration polarization and non-ideal gas behavior. Concentration polarization is the phenomenon of reduced concentration near the membrane surface that results in reduced effective permeance due to non-idealities arising from the convective and diffusive transport. Several modeling studies have explored these effects, with the requirement of a 2D or 3D geometry to model the diffusion effects perpendicular to the convective flow [12,13,14]. Concentration polarization is increasingly significant at higher permeances, but some techniques can reduce the effects. Feed spacers or baffles aid in gas mixing within the membrane module [15,16,17,18], showing that some of the non-ideal geometry issues can be solved by optimizing the design of the separator itself. In the case of propylene/propane separation, it has been shown that modified equations of state were necessary to correctly calculate gas properties and accurately model the membrane separator [19]. Nevertheless, a large number of studies have shown that simplified 1D models with ideal gas assumptions can accurately model some gas separations [1,9,10], making them efficient for techno-economic studies and surveys of large variable spaces due to the low computational power required. Two recent reviews by Kancherla et al. [20] and Foo et al. [21] on the topic of membrane separator modeling provide thorough overviews of the aforementioned topics.

Optimization of a membrane separator can involve either direct operational parameters, typically purity and recovery, or cost targets, such as total membrane area and compression costs. Due to limitations with purity and recovery in single-stage separators, techno-economic assessments rely on multi-stage separators with recycle loop configurations to obtain the cheapest potential membrane separations [1,22,23,24,25,26]. Amongst these studies, several techno-economic designs suggest operating at pressure ratios of less than 50 [25,26,27,28,29]. However, low pressure ratios combined with high recovery targets can lead to limitations in the obtainable purity. Previously, Kaldis et al. [30] observed a notable lack of purity or recovery increase with increasing membrane selectivity when a membrane separator was modeled at a pressure ratio of 20. Similarly, a fractionally decreasing cost reduction was reported for increasing selectivity above ca. 30 by Ahmad et al. [23]. However, neither study explored the limited purity in detail. Huang et al. [31] introduced the concept of a pressure-ratio-limited regime, in which operating below a limiting feed concentration results in the purity being limited by the pressure ratio. Yet, while this study offered insight into the maximum purity trends, it did not provide any equations for predicting the pressure-ratio-limited regime without modeling each condition explicitly. In the current work, a membrane separator model is used to explore the parameter space of operation across a much wider range than previously reported, with an emphasis on the purity limitations of membrane separators and the resultant useful membrane selectivity targets.

## 2. Materials and Methods

### 2.1. Model Assumptions

A simplified membrane-based gas separator model has been employed with the following assumptions: (1) steady-state operation; (2) isothermal operation; (3) isobaric operation; (4) negligible dispersion (diffusion) effects; and (5) constant permeance and selectivity.

For continuous membrane separation processes, a system can be assumed to be running at a steady state such that no time dependencies remain. Because the permeation process involves negligible energy changes (e.g., adsorption/desorption), an isothermal assumption is valid. The isobaric assumption is valid for larger geometry membranes such as supported silica or zeolite membranes on porous supports with radii greater than ca. 5 mm but is less valid for spiral-wound or densely-packed hollow fiber polymer membrane modules. Conversely, the negligible dispersion assumption is more valid for spiral-wound or hollow fiber modules and less valid for larger supported membrane modules due to the increased radial distance of supported membrane tubes. In both cases, the assumptions imply an idealized system, with decreases in performance expected in real systems. However, techno-economic assessments commonly employ a simple 1D approach with isobaric conditions to obtain approximate notions of the economic feasibility of a membrane process [22,26]. Lastly, the use of constant permeance and selectivity means that adsorption-induced permeance inhibitions or time-dependent degradations in the membrane structure are ignored. The effects of permeance-inhibitions can be ignored because the modeled permeance can be considered an effective permeance rather than the theoretical permeance of a specific material. The use of theoretical permeance would be applicable when optimizing a specific membrane system, but the goal is to obtain a generalized result that is independent of membrane selection so that the general trends of the membrane separator can be understood. Note, however, that this effective permeance does not include concentration polarization effects, which would require a 2D model to accurately interpret.

### 2.2. Membrane Geometry and Solving Environment

**Figure 1:**
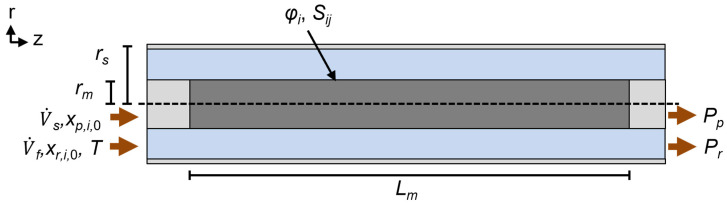
Schematic of a shell-and-tube membrane separator with relevant geometric and operational parameters for modeling purposes. Blue: retentate gas phase, gray: permeate gas phase (light) and membrane (dark). Note that the sweep flow will be assumed negligible in the final system, so its placement as co-current or counter-current is irrelevant.

The geometry of the membrane separator is assumed to be a concentric shell-and-tube design with a membrane placed in the inner volume of an outer shell, the feed introduced on the shell side, and the permeate collected on the lumen side. For a single tube separator, this is shown schematically in Figure 1 with the required variables to define the system labeled and tabulated (Table 1). The geometry is defined by the membrane length, *L_m_* [m], membrane radius, *r_m_* [m], and shell radius, *r_s_* [m]. The membrane properties include the permeance of species *i*, *φ_i_* [mol m^−2^ s^−1^ Pa^−1^], and the ideal selectivity of species *i* vs. *j*, *S_ij_*. Lastly, the operational conditions include the feed temperature, *T* [K], the feed composition of each species *i*, *x_r_*_,*i*,0_, the total volumetric feed flow rate, $\dot{V}$*_f_* [m^3^ s^−1^], the retentate pressure, *P_r_* [Pa], the sweep composition of each species *i*, *x_p_*_,*i*,0_, the total volumetric sweep flow rate, $\dot{V}$*_s_* [m^3^ s^−1^], and the permeate total pressure, *P_p_* [Pa]. Although this system is for a single tube, the final model derivation allows for the comparison of differing geometries via normalization of the active permeable membrane area and total volume on the retentate side. As will be shown in the derivation, the dimensional analysis results in a grouping of terms for the geometry such that only the membrane area *A_m_* [m^2^] remains. Furthermore, although the model shows a co-current flow, the presence of the sweep is for model convergence only and the final results can be assumed to occur for systems with zero sweep flow.

This model has been developed in the finite element analysis software COMSOL Multiphysics^®^ 6.1 (Burlington, MA, USA). The retentate and permeate transport equations were solved simultaneously using a parallel sparse direct solver (PARDISO) algorithm. A mesh consisting of 100 domain elements was used for the iterative solver and the solution was determined based on residual or error criteria below 10^−6^.

### 2.3. Model Equations

#### 2.3.1. Dimensional Mass Balances and Variable Transformations

The governing steady-state transport equations with the aforementioned assumptions for the retentate and permeate streams of a 1D membrane separator model are as follows:

(1) $$u_{r,z}\frac{\partial c_{r,i}}{\partial z}=\frac{-P_{m}J_{i}}{A_{r}}$$

(2) $$u_{p,z}\frac{\partial c_{p,i}}{\partial z}=\frac{P_{m}J_{i}}{A_{p}}$$

where *u_z_* [m s^−1^] is the velocity in the axial direction, *c_i_* [mol m^−3^] is the bulk concentration of species *i*, *J_i_* [mol m^−2^ s^−1^] is the flux of species *i* through the membrane, *P_m_* [m] is the circumference of the membrane, *A_r_* [m^2^] is the cross-sectional area of the retentate, *A_p_* [m^2^] is the cross-sectional area of the permeate, and the subscripts r and p denote the retentate and permeate sides, respectively. A negative flux is assigned to the retentate, and a positive flux is assigned to the permeate to balance the species leaving and entering each system, respectively. Within the COMSOL solving environment, the 1D model requires definitions for a physical system including all of the parameters listed except for *r_s_*.

The concentrations are related to mole fractions using the ideal gas relationship, as follows:

(3) $$c_{r,i}=\frac{x_{r,i}P_{r}}{RT}$$

(4) $$c_{p,i}=\frac{x_{p,i}P_{p}}{RT}$$

where *R* [J mol^−1^ K^−1^] is the universal gas constant and *T* [K] is the temperature. To simplify the model and reduce variable dependence, dimensionless variables were introduced. The axial length was reduced using the following:

(5) $$\zeta =\frac{z}{L_{m}}$$

where *L_m_* [m] is the length of the membrane. The flow velocity was reduced with the following:

(6) $$\upsilon_{r,z}=\frac{u_{r,z}}{u_{r,z,0}}$$

(7) $$\upsilon_{p,z}=\frac{u_{p,z}}{u_{p,z,0}}$$

where *u_r_*_,*z*,0_ [m s^−1^] is the inlet velocity of the feed, and *u_p,z,_*_0_ [m s^−1^] is the inlet velocity of the sweep. The feed and sweep velocities are converted to volumetric feed flow rates using the following:

(8) $$u_{r,z,0}=\frac{{\dot{V}}_{f}}{A_{r}}$$

(9) $$u_{p,z,0}=\frac{{\dot{V}}_{s}}{A_{p}}$$

where $\dot{V}$*_f_* [m^3^ s^−1^] is the feed flow rate and $\dot{V}$*_s_* [m^3^ s^−1^] is the sweep flow rate. The volumetric flow rates are converted to standard flow rates $\dot{V}$*_f_*_,STP,_ and $\dot{V}$*_s_*_,STP_ assuming ideal gas relationships, as follows:

(10) $${\dot{V}}_{f}=\frac{P_{0}T}{T_{0}P_{r}}{\dot{V}}_{f,\mathrm{STP}}$$

(11) $${\dot{V}}_{s}=\frac{P_{0}T}{T_{0}P_{p}}{\dot{V}}_{s,\mathrm{STP}}$$

where *P*_0_ is the standard pressure [100 kPa] and *T*_0_ is the standard temperature [273.15 K]. Lastly, permeance through the membrane, *φ_i_* [mol m^−2^ s^−1^ Pa^−1^], is modeled as a pressure-driven flux gradient with the following:

(12) $$\varphi_{i}=\frac{J_{i}}{\left(x_{r,i}P_{r}-x_{p,i}P_{p}\right)}$$

where *x_r_*_,*i*_ is the local retentate mole fraction of species *i*, and *x_p_*_,*i*_ is the local permeate mole fraction of species *i*. Relative permeances of differing species are related by the ideal selectivity of species *i* vs. *j*, *S_ij_*, using the following:

(13) $$S_{ij}=\frac{\varphi_{i}}{\varphi_{j}}.$$

Combining the previous equations, substituting into the mass balances, and rearranging terms results in the following:

(14) $$\upsilon_{r,z}\frac{\partial x_{r,i}}{\partial \zeta }=\frac{-RT_{0}A_{m}\varphi_{i}P_{r}}{{\dot{V}}_{f,\mathrm{STP}}P_{0}}\left(x_{r,i}-x_{p,i}\frac{P_{p}}{P_{r}}\right)$$

(15) $$\upsilon_{p,z}\frac{\partial x_{p,i}}{\partial \zeta }=\frac{RT_{0}A_{m}\varphi_{i}P_{r}}{{\dot{V}}_{s,\mathrm{STP}}P_{0}}\left(x_{r,i}-x_{p,i}\frac{P_{p}}{P_{r}}\right)$$

where *A_m_* [m^2^] is the membrane permeable area. Note that in this form the geometric terms for the tubular geometry have disappeared and only *A_m_* remains. From this, it is clear that any geometry (e.g., planar or tubular) can be used, as well as any multilayer or multitube system. As long as the separator acts as a single stage (i.e., a single feed, single retentate, and single permeate stream), only the effective total membrane surface area is required to model the system.

#### 2.3.2. Dimensionless Transport Parameter and Reduced Pressure

From the dimensionless forms of the mass balances, two dimensionless parameters are observed. The first will be called a transport variable, *θ_i_*, as follows:

(16) $$\theta_{i}=\frac{{\dot{V}}_{f,\mathrm{STP}}P_{0}}{RT_{0}A_{m}\varphi_{i}P_{r}}$$

which balances the feed flow rate on the numerator with the membrane permeance, membrane area, and retentate pressure on the denominator. The transport parameter is defined for each species *i*, but note that each *θ_i_* is related to other species using *S_ij_* because the only difference in the definition is *φ_i_*. Variants of this parameter have been used in other dimensionless approaches to membrane reactors [32], sometimes being referred to as a modified Pe number by [33]. The second relationship is the reduced pressure, *ψ*, as follows:

(17) $$\psi =\frac{P_{r}}{P_{p}}$$

where *P_r_* is placed in the numerator such that, intuitively, as *ψ* increases, so does the permeation driving force in the membrane system. This definition, or its inverse, is used often in modeling studies to relate the retentate and permeate pressures of a membrane system.

#### 2.3.3. Dimensionless Mass Balances

Upon replacement of terms in the mass balances with the dimensionless groups defined here, the final mass balances can be written as follows:

(18) $$\upsilon_{r,z}\frac{\partial x_{r,i}}{\partial \zeta }=\frac{-\left(\psi x_{r,i}-x_{p,i}\right)}{\theta_{i}\psi }$$

(19) $$\upsilon_{r,z}\frac{\partial x_{r,j}}{\partial \zeta }=\frac{-S_{ij}\left(\psi x_{r,i}-x_{p,i}\right)}{\theta_{i}\psi }$$

(20) $$\upsilon_{p,z}\frac{\partial x_{p,i}}{\partial \zeta }=\frac{{\dot{V}}_{f,\mathrm{STP}}}{{\dot{V}}_{s,\mathrm{STP}}}\frac{\left(\psi x_{r,i}-x_{p,i}\right)}{\theta_{i}\psi }$$

(21) $$\upsilon_{p,z}\frac{\partial x_{p,j}}{\partial \zeta }=\frac{{\dot{V}}_{f,\mathrm{STP}}}{{\dot{V}}_{s,\mathrm{STP}}}\frac{S_{ij}\left(\psi x_{r,i}-x_{p,i}\right)}{\theta_{i}\psi }$$

where the sweep side includes the ratio of feed to sweep flow rates at the inlet. Based on this definition, a zero sweep condition results in an unstable system so $\dot{V}$*_s_* was set to be 0.001% of $\dot{V}$*_f_*. For all conditions modeled, the sweep flow remained negligibly low at less than 0.1% of the permeated flow when the recovery was greater than 1%. The use of negligible permeate sweep flow means that the steady-state permeate concentrations will be constant along the length of the module. This causes the results to be identical whether the system is modeled as a co-current or counter-current configuration. Results discussed herein apply to systems with zero sweep flow such that the maximum potential permeate purity can be achieved. Due to the introduction of the reduced flow velocity parameter, the flow rate boundary conditions at the inlets are as follows:

(22) $${\left.\upsilon_{r,z}\right|}_{z=0}=1$$

(23) $${\left.\upsilon_{p,z}\right|}_{z=0}=1.$$

The inlet concentrations of both retentate and permeate sides must be specified for n − 1 species, where n is the total number of species, as follows:

(24) $${\left.x_{r,i}\right|}_{z=0}=x_{r,i,0}$$

(25) $${\left.x_{p,i}\right|}_{z=0}=x_{p,i,0}.$$

Because a negligible sweep flow is introduced the inlet permeate compositions do not affect the results, so the values were set arbitrarily to an equimolar concentration to prevent poor convergence of the model. In total, the model requires 2 dimensionless inputs (*θ_i_*, *ψ*), n − 1 selectivities (*S_ij_*), and n − 1 inlet feed compositions (*x_r_*_,*i*,0_) to be defined, resulting in a total requirement of 2n input parameters to specify a 1D membrane separator (Table 2). To keep the number of variables reasonable, a binary gas mixture is assumed on the basis that tertiary-order gases can be classified as either low permeance (i.e., high *S_ij_*) or low feed concentration compared to the primary two gases. Thus, a system consisting only of gases *i* and *j*, and requiring 4 input criteria was used for further analysis.

#### 2.3.4. Performance Criteria

The goal of this study is to elucidate the maximum separation capability of a membrane separator in terms of total flow productivity, recovery, and purity. Recovery of species *i* is defined as follows:

(26) $$R_{i}=\frac{{\dot{n}}_{r,i,0}-{\dot{n}}_{r,i,1}}{{\dot{n}}_{r,i,0}}$$

where *ṅ_r_*_,*i*,0_ is the inlet molar flow rate of species *i* and *ṅ_r_*_,*i*,1_ is the outlet molar species of *i* in the retentate. Purity is defined as follows:

(27) $$x_{p,i}=\frac{{\dot{n}}_{p,i}}{{\dot{n}}_{p}}$$

where *ṅ_p_*_,*i*_ is the outlet molar flow rate of species *i*, and *ṅ_p_* is the total outlet molar flow rate. A maximum volumetric productivity can be acquired, for example, by setting targets for *R_i_* or *x_p_*_,*i*_ and obtaining the highest value of *θ_i_* possible.

## 3. Results

### 3.1. Modified Pressure Ratio

In the derivations, the total pressure ratio (*P_r_*/*P_p_*) was used to define *ψ*, which is reasonable given its appearance in the mass balances. However, a simple ratio of pressures fails to account for the change in partial pressures of the system, particularly when the species of interest is at a low concentration in the inlet. Instead, a reduced pressure ratio, *ψ_i_*, based on the permeance equation to balance the partial pressure gradient across the membrane is required, as follows:

(28) $$\psi_{i}=\frac{x_{r,i,0}P_{r}}{x_{p,i,0}P_{p}}\approx \frac{x_{r,i,0}P_{r}}{P_{p}}.$$

This ratio is a measure of the effective driving force for permeation at the separator inlet, as it uses the feed and sweep inlet concentrations (*x_r_*_,*i*,0_ and *x_p_*_,*i*,0_). At a value of unity the driving force for permeation of species *i* is zero so no separation occurs, and as *ψ_i_* increases the driving force increases resulting in higher recovery. Because the sweep gas in this system is negligibly small to avoid issues with further purification, the value of *x_p_*_,*i*,0_ is not a known input. However, for conditions at which *φ_i_*Δ*P_i_*_,0_ ≫ *φ_j_*Δ*P_j_*_,0_ the value of *x_s_*_,*i*,0_ converges on unity, which will be increasingly valid as *S_ij_* increases. Thus, a value of *x_p_*_,*i*,0_ = 1 is used for all conditions. Importantly, this decision does not affect the accuracy of the results as it only changes the input parameter calculations, not the model equations themselves. Previously, Huang et al. [31] discussed a similar modified pressure ratio.

**Figure 2:**
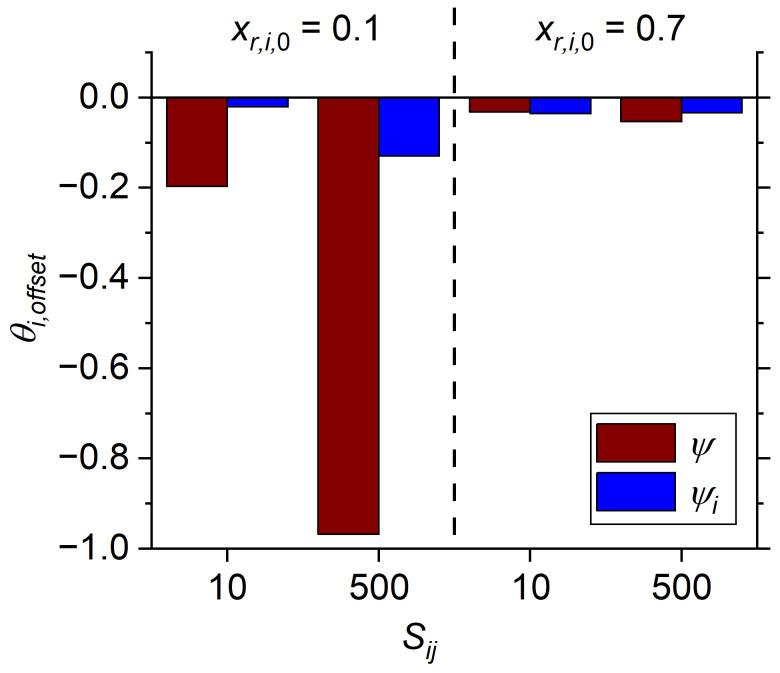
Offset in *θ_i_* between the unmodified *ψ* and modified *ψ_i_* ratios at low and high *x_r_*_,*i*,0_, and low and high *S_ij_* conditions. Offset is calculated as the difference between *θ_i_* at either *ψ_i_* = 10^2^ and 10^4^ or *ψ* = 10^2^ and 10^4^ with *R_i_* of 98%.

The ability of the modified *ψ_i_* term to normalize pressure driving force is illustrated in Figure 2 using a *θ_i_*_,*offset*_ value defined as follows:

(29) $$\theta_{i,offset}=\frac{{\left.\theta_{i}\right|}_{R_{i}=0.98,\psi_{i}=10^{2}}-{\left.\theta_{i}\right|}_{R_{i}=0.98,\psi_{i}=10^{4}}}{{\left.\theta_{i}\right|}_{R_{i}=0.98,\psi_{i}=10^{4}}}.$$

In this equation, the value of *θ_i_*_,*offset*_ is determined as a fractional difference between the *θ_i_* obtained for 98% recovery at a *ψ_i_* of 10^2^ and 10^4^. Equivalently, a *θ_i_*_,*offset*_ is also calculated using the unmodified *ψ* of 10^2^ and 10^4^ for comparison. The *θ_i_*_,*offset*_ was calculated at *x_r_*_,*i*,0_ of 0.10 and 0.70, and *S_ij_* of 10 and 500, and shown for both *ψ* and *ψ_i_* in Figure 2. At higher *x_r_*_,*i*,0_ the *θ_i_*_,*offset*_ is low and similar for both *ψ* and *ψ_i_*, which is reasonable as the value of *ψ* and *ψ_i_* converge at a feed mole fraction of one. However, at *x_r_*_,*i*,0_ = 0.10 the *θ_i_*_,*offset*_ is significantly more negative when calculated using *ψ*, meaning that increasing the pressure ratio from 10^2^ to 10^4^ would result in a significant increase in the obtained *θ_i_* (i.e., higher productivity with higher pressure gradient). The use of *ψ_i_* compresses the change in *θ_i_* with respect to *x_r_*_,*i*,0_ and *S_ij_*, and Figure S1 indicates that negligible change in *R_i_* is expected above *ψ_i_* of 10^3^ for any condition tested (*x_r_*_,*i*,0_ > 0.10 and *S_ij_* < 500).

### 3.2. Absolute Maximum Permeate Purity

**Figure 3:**
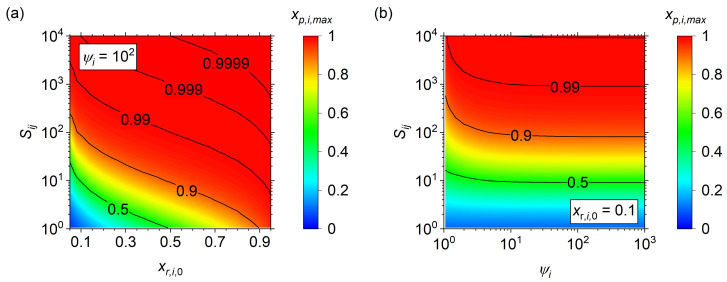
Maximum purity, *x_p,i,max_*, of an ideal membrane separator using an analytical solution (*R_i_* = 0). (**a**) *x_p_*_,*i*,*max*_ for *S_ij_* vs. *x_r,i,0_* at *ψ_i_* = 10^2^, (**b**) *x_p_*_,*i*,*max*_ for *S_ij_* vs. *ψ_i_* at *x_r_*_,*i*,0_ = 0.10.

The absolute maximum purity of the membrane separator is obtained as an analytical solution for the limiting case in which the recovery (stage cut) goes to zero [19], given as follows:

(30) $$x_{p,i,\max}={\left.x_{p,i}\right|}_{\lim R_{i}\to 0}=\frac{\psi }{2}\left(x_{r,i,0}+\frac{1}{\psi }+\frac{1}{S_{ij}-1}-\sqrt{{\left(x_{r,i,0}+\frac{1}{\psi }+\frac{1}{S_{ij}-1}\right)}^{2}-\frac{4S_{ij}x_{r,i,0}}{\psi \left(S_{ij}-1\right)}}\right)$$

which requires *x_r_*_,*i*,0_, *S_ij_*, and *ψ* to be defined. The maximum purity, *x_p_*_,*i*,*max*_, is shown for *S_ij_* vs. *x_r_*_,*i*,0_ at a *ψ_i_* of 10^2^ in Figure 3a. Both *S_ij_* and *x_r_*_,*i*,0_ strongly influence *x_p_*_,*i*,*max*_, with the *x_p_*_,*i*,*max*_ converging on *x_r_*_,*i*,0_ as *S_ij_* decreases to unity. *S_ij_* = 1 implies the membrane is unselective and all species transport equally, so the purity is equal to the input concentration. Generally, *x_p_*_,*i*,*max*_ increases as either *x_r_*_,*i*,0_ or *S_ij_* increases. Next, *x_p,i,max_* is shown for *S_ij_* vs. *ψ_i_* at *x_r_*_,*i*,0_ of 0.10 in Figure 3b, wherein the majority of change with *ψ_i_* occurs for *ψ_i_* < 10. Above *ψ_i_* = 10, the maximum purity becomes asymptotic with little change in the range of *S_ij_* explored. However, note that the change in *x_p_*_,*i*,*max*_, with respect to *ψ_i_*, increases as *S_ij_* increases, indicating a substantially high *S_ij_* would require an increased *ψ_i_* to stabilize (i.e., *ψ_i_* > 10^2^ at *S_ij_* > 10^4^).

### 3.3. The Effect of Recovery on the Maximum Purity

**Figure 4:**
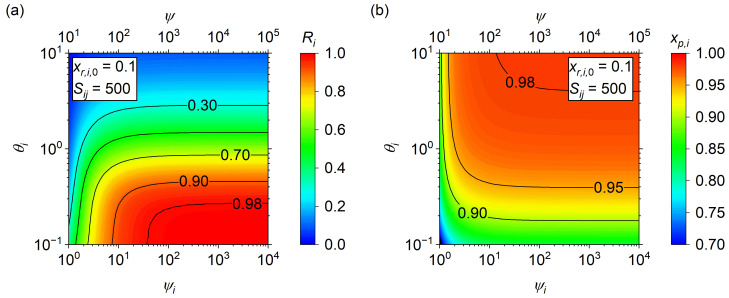
(**a**) Product recovery, *R_i_*, and (**b**) permeate purity, *x_p_*_,*i*_, dependence on the transport parameter, *θ_i_*, and modified dimensionless pressure ratio *ψ_i_* at *x_r_*_,*i*,0_ = 0.10 and *S_ij_* = 500.

The issue with the analytical solution, with regard to maximum purity, is that it only applies at *R_i_* = 0. To understand the effects of increasing recovery on the maximum attainable purity, first consider the effects of *θ_i_* and *ψ_i_* on *R_i_* (Figure 4a) and *x_p_*_,*i*_ (Figure 4b). Although increasing *ψ_i_* results in an asymptotic *R_i_* and *x_p_*_,*i*_, the contour lines arise from opposite ends of the *θ_i_* scale, which means the maximum *x_p_*_,*i*_ at a specified *R_i_* occurs at the highest *ψ_i_* possible. As before, the majority of change occurs for *ψ_i_* < 10^2^, so a value of *ψ_i_* = 10^2^ will be chosen as a reasonable maximum operating condition. Note that, as previously mentioned and shown in ESI Figure S1, the selection of *ψ_i_* = 10^2^ is reasonable across a wide range of *x_r_*_,*i*,0,_ and *S_ij_*. Furthermore, it is important to realize that real systems will not likely operate at *ψ_i_* > 10^2^, but the choice of pressure ratio is driven by the desire to obtain an estimate of the maximum potential purity. Any decrease in pressure ratio driven by economic arguments will result in a lower obtained purity, which is the reason this result may be considered the maximum obtainable purity of a single-stage separator.

**Figure 5:**
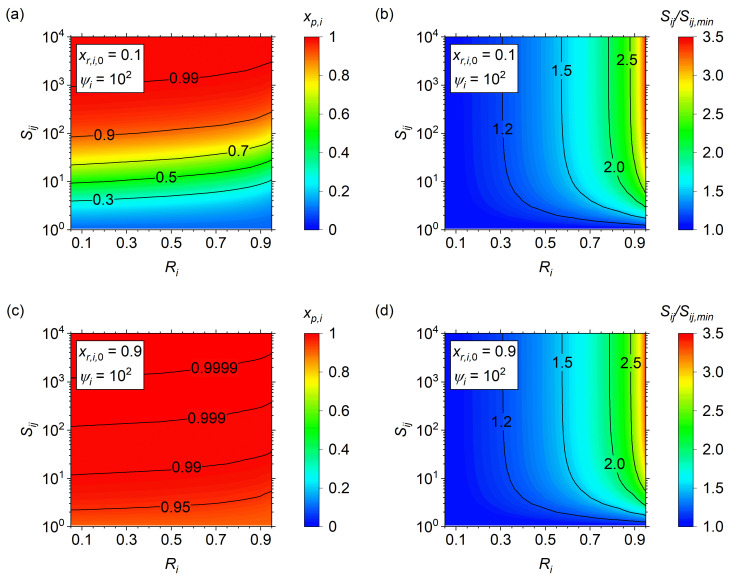
Maximum purity of the 1D model (**a**,**c**) and relative selectivity vs. the analytical solution (**b**,**d**) vs. *S_ij_* and *R_i_* for (**a**,**b**) *x_r_*_,*i*,0_ = 0.10, *ψ_i_* = 10^2^, and (**c**,**d**) *x_r_*_,*i*,0_ = 0.90, *ψ_i_* = 10^2^.

Using *ψ_i_* = 10^2^ as the pressure ratio, the effects of *S_ij_* and *R_i_* on the maximum purity are shown for *x_r_*_,*i*,0_ of 0.10 (Figure 5a) and 0.90 (Figure 5c). Here, the maximum purity was obtained using simulated data by optimizing *θ_i_* until the target *R_i_* was achieved for each combination of *S_ij_*, *x_r_*_,*i*,0_, and *ψ_i_*. As *R_i_* increases, *x_p_*_,*i*_ slightly decrease for a specified *S_ij_*. Furthermore, the contour lines show that while the absolute *x_p_*_,*i*_ changes with *x_r_*_,*i*,0_, the overall trends of constant *x_p_*_,*i*_ remain similar. To illustrate this effect in detail, the *S_ij_* at a specified *R_i_* was compared to the analytical solution *S_ij_* as *R_i_* goes to zero (*S_ij_*_,*min*_), and shown as surface plots in Figure 5b,d. This information can be useful in predicting the minimum selectivity of a separation given a target *R_i_* and *x_p_*_,*i*_ using the relationship

(31) SijSij,min=Sijxp,i,RiSijxp,i,Ri=0.

### 3.4. Estimating the Minimum Selectivity for a Desired Purity and Recovery

Although the analytical result of Equation (30) can be solved to obtain a selectivity target for a desired purity, the change with recovery is not considered. Using the relationship in Equation (31) and data from Figure 5, an improved estimate for the minimum selectivity can be obtained via the following:

1. Using the analytical solution (Equation (30)), solve for the *S_ij_* required to obtain the target *x_p_*_,*i*_ at the specified *x_r_*_,*i*,0_, and use a *ψ_i_* > 10^2^. Consider this *S_ij_* as *S_ij_*_,*min*_;
2. Use the relationship shown in Figure 5b to identify the multiplication factor for the target *R_i_* (e.g., 2.7 at *R_i_* = 90%, *S_ij_* of 10^2^);
3. Multiply the *S_ij_*/*S_ij_*_,*min*_ factor by *S_ij_*_,*min*_ to obtain the minimum selectivity required to achieve the target *x_p,i_* at the target *R_i_*.

Using these steps, the application is explained using separation of CO_2_ for carbon capture projects; however, note that the method can be applied to higher selectivity operations or differing feed concentrations depending on the target application. CO_2_ separation membranes have a typical *S_ij_* range of less than 100, with a maximum no higher than ca. 800 [34,35,36]. Sources of CO_2_ include flue gas from burners, with low CO_2_ concentrations of about 10% being common [11,37]. Thus, a 10% CO_2_ feed is considered, and the discussion will focus on identifying *S_ij_* targets of 50 to 100 as reasonable. Table 3 shows the results for the example separation of 10% CO_2_ for carbon capture applications. The result of solving Equation (30) is provided as *R_i_* = 0% and then targets of 90% and 95% recovery are set to show the difference. In this sample, the target purity of 90% is seemingly achievable for CO_2_ membranes using Equation (30), with a required *S_ij_* of 89. However, raising the *R_i_* to 95% shows that the true minimum *S_ij_* is 300 for the high recovery targets of carbon capture, which is in the upper range of all reported CO_2_ membranes [34,35,38]. Instead, a more accurate maximum final purity will be closer to 70%, which shows that a recovery of 95% may be achieved with a selectivity of 72. Note that this minimum *S_ij_* is still an underestimation due to the expected overprediction of both high *ψ_i_* and the 1D model in general, but the outcome is simple to calculate and can provide a significantly more accurate target *S_ij_* than the analytical solution at *R_i_* = 0% alone.

### 3.5. Limiting Pressure Ratios and Asymptotic Purity Zones

**Figure 6:**
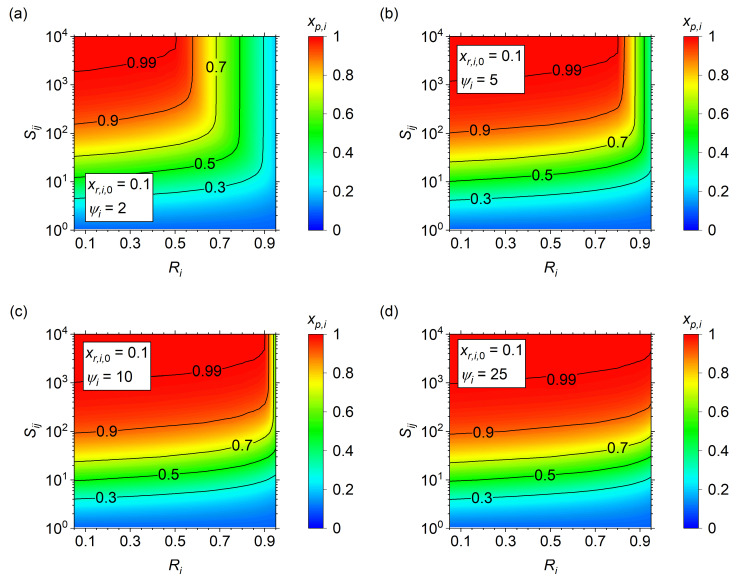
Resultant *x_p_*_,*i*_ surface plots for *S_ij_* vs. *R_i_* at *x_r_*_,*i*,0_ of 0.10 and (**a**) *ψ_i_* = 2, (**b**) *ψ_i_* = 5, (**c**) *ψ_i_* = 10, and (**d**) *ψ_i_* = 25.

Although the previous section provides insight into the minimum selectivity at high recovery, the primary limitation is that it applies only to high pressure ratios that are almost certainly not economically viable [26,29]. To explore the effects at lower pressure ratios, *x_p_*_,*i*_ is plotted against *S_ij_* vs. *R_i_* for pressure ratios of *ψ_i_* = 2, 5, 10, and 25 in Figure 6. At *ψ_i_* = 2 (Figure 6a), *x_p_*_,*i*_ increases slowly with *R_i_* until an asymptotic rise is observed. The asymptotic *R_i_* value increases with increasing *S_ij_*, but poses a severe limitation on the maximum achievable purity at high recovery for all *S_ij_* of interest. Increasing *ψ_i_* to 5 (Figure 6b), the asymptotic rise is delayed to higher *R_i_*, but is still a concern at *R_i_* > 0.8 for the *S_ij_* up to 10^4^. Further increasing *ψ_i_* to 10 (Figure 6c) increases the limiting Ri to about 0.90, but it requires an increase in *ψ_i_* to 25 (Figure 6d) to remove the limiting regime up to the maximum plotted condition of *R_i_* = 0.95. Note that the asymptote arises due to the physical limitations imposed by permeation at near-complete recovery. As *R_i_* goes to unity, *θ_i_* must go to zero to allow for 100% of the inlet target species to permeate due to the increase in residence time required for permeation. This means that the asymptote will arise for all *ψ_i_*, but at sufficiently high *ψ_i_*, this situation does not occur until the *R_i_* is above a reasonable target and can be ignored.

The important lesson from Figure 6 is that at high *R_i_* and low *ψ_i_*, the asymptotic trends result in a maximum effective *S_ij_*. Increasing *S_ij_* beyond the maximum results in no increase in purity and is unlikely to be economically viable considering higher *S_ij_* is associated with lower membrane permeance (productivity) and higher development and fabrication costs. Thus, identifying the regime in which the asymptotic maximum *S_ij_* arises is important in understanding the target regions of operation.

### 3.6. Obtention of the Limiting Selectivity

Although the information presented in Figure 6 could be construed as a maximum *R_i_* for a given set of conditions, it is more practical to consider a minimum *S_ij_* to avoid asymptotic regions. To approach this, first consider the residual impurity

(32) $$\xi_{p,i}=1-x_{p,i}.$$

**Figure 7:**
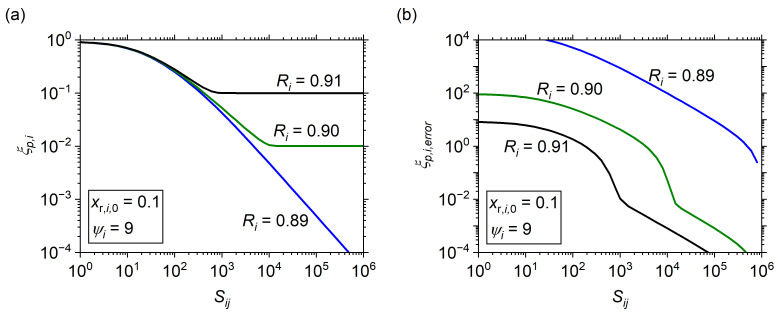
(**a**) Impurity level vs. *S_ij_*, and (**b**) impurity error vs. *S_ij_* for *R_i_* of 0.89, 0.90, and 0.91 at *x_r_*_,*i*,0_ of 0.10 and *ψ_i_* of 9.

The use of impurity is for convenience; a plot of the impurity as the value goes to zero is observed easily on a log scale compared to purity with its convergence on unity. Figure 7a shows sample data of the impurity level vs. *S_ij_* at *x_r_*_,*i*,0_ of 0.10, *ψ_i_* of 9, and *R_i_* that straddle the asymptotic region. Note that *ψ_i_* of 9 is an arbitrary choice, but it clearly illustrates the asymptotic effect that can occur near a target recovery of 90%; any value of *ψ_i_* sufficiently low as to have an asymptotic purity regime could have been used, with appropriate change in *R_i_* to illustrate the asymptotic phenomena. At *R_i_* of 0.89, the impurity level decreases continuously as *S_ij_* increases toward 10^6^, whereas the impurity level plateaus for *R_i_* of 0.90 and 0.91. The plateau for *R_i_* of 0.90 occurs at *S_ij_* of ca. 10^4^, whereat further increases in *S_ij_* do not decrease the impurity level below ca. 0.01 (*x_p_*_,*i*_ = ca. 0.99). The effect is worse at *R_i_* of 0.91, with a minimum impurity level of ca. 0.1 (*x_p_*_,*i*_ = ca. 0.9) at *S_ij_* of ca. 10^3^.

To obtain an approximate *S_ij_* at which the impurity level no longer declines, an error is defined as follows:

(33) $$\xi_{p,i,error}=\left|\frac{\xi_{p,i}-{\left.\xi_{p,i}\right|}_{S_{ij}=10^{8}}}{{\left.\xi_{p,i}\right|}_{S_{ij}=10^{8}}}\right|.$$

where the difference between the impurity level at some specified *S_ij_* and at *S_ij_* = 10^8^ is divided by the level at *S_ij_* = 10^8^. A maximum selectivity of 10^8^ was defined because it is sufficiently close to infinity when considering that thin film membrane technologies do not achieve selectivity greater than 10^5^ even for the best-reported composite Pd membranes [39] (except for those in which the true impurity was below the detection limit of the equipment and the authors mistakenly claim infinity). The results of this calculation with respect to the conditions of Figure 7a are shown in Figure 7b. For the *R_i_* = 0.89 condition, the error decreases continuously but never drops below 10^−2^ at *S_ij_* less than 10^6^. Contrastingly, the two *R_i_* with asymptotic impurity levels both experience a rapid decline to below 10^−2^ before declining further. Given this information, a change of 10^−2^ is chosen arbitrarily as a sufficient predictor of the asymptotic *S_ij_* value, as it suggests that any further increase in *S_ij_* will result in less than a 1% further increase in purity. Using the technique developed above, the maximum selectivity with continued decreases in impurity level was obtained across the parameter space by performing two steps. First, *x_p,i_* data were obtained for a range of *S_ij_* at a specified *x_r_*_,*i*,0_, *ψ_i,_* and *R_i_* (*R_i_* is obtained by optimizing *θ_i_*). Second, *ξ_p_*_,*i*_ and *ξ_p_*_,*i*,*error*_ were calculated for each data point, and then *S_ij_* that results in *ξ_p_*_,*i*,*error*_ = 10^−2^ was interpolated, up to the maximum *S_ij_* = 10^8^ range used; this *S_ij_* value is defined as *S_ij_*_,*max*_.

**Figure 8:**
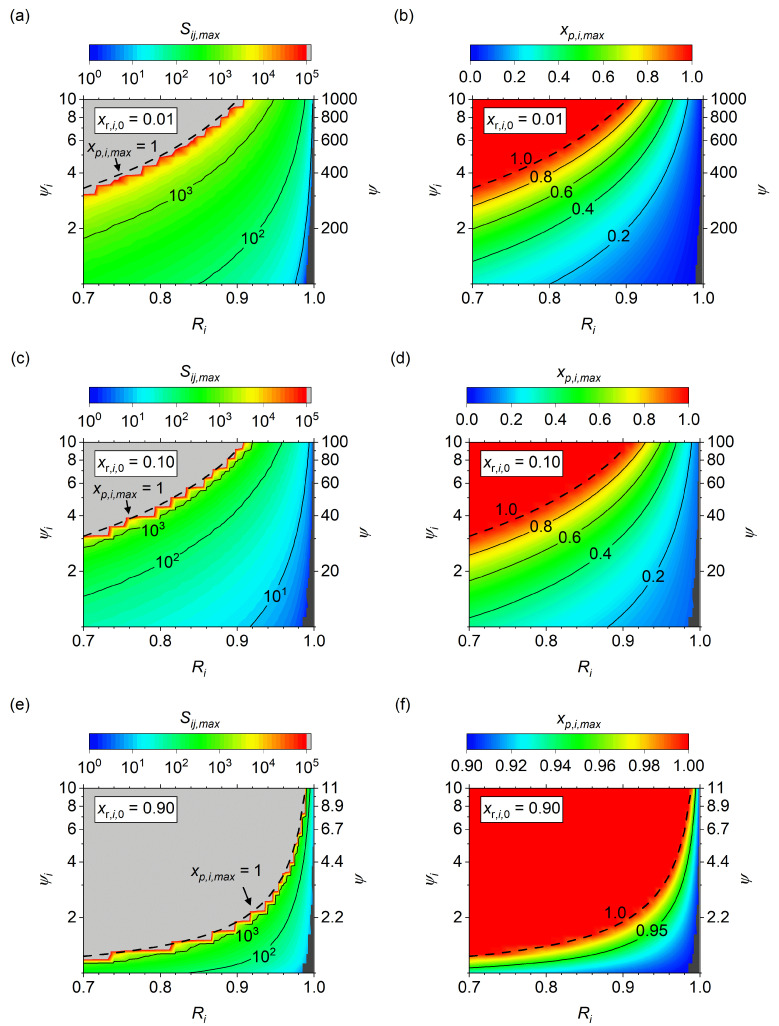
*S_ij_*_,*max*_ and *x_p_*_,*i*,*max*_ surface plots of *ψ_i_* vs. *R_i_* at *x_r_*_,*i*,0_ of 0.01 (**a**,**b**), 0.10 (**c**,**d**), and 0.90 (**e**,**f**). Unmodified pressure ratio, *ψ*, labeled on the right axis for comparison. Light gray zones indicate no asymptotic purity observed up to the maximum simulated *S_ij_* of 10^8^. Solid lines represent *S_ij,max_* or *x_p,i,max_* contours. The dashed line *x_p_*_,*i*,*max*_ = 1 originates from Equation (33). Roughness in data near the dashed line due to discrete data point fidelity and the rapid rise toward infinite *S_ij_*.

The results of the fitting process are shown as surface plots of *S_ij_*_,*max*_ and *x_p_*_,*i*,*max*_ across *ψ_i_* vs. *R_i_* for *x_r_*_,*i*,0_ of 0.01 (Figure 8a,b), *x_r,i,_*_0_ of 0.10 (Figure 8c,d), *x_r_*_,*i*,0_ of 0.90 (Figure 8e,f). First, consider the case of *S_ij_*_,*max*_ for *x_r_*_,*i*,0_ of 0.01 (Figure 8a). *S_ij_*_,*max*_ increases with increasing *ψ_i_* but decreasing *R_i_*. Due to the rapid approach toward infinite *S_ij_*, an arbitrary maximum *S_ij_* of 10^5^ was used for clarity in the plot, with the gray-colored region effectively infinity (i.e., *S_ij_* > 10^8^). Note that while the modified pressure ratio (*ψ_i_*) varies between 1 and 10, the absolute pressure ratio (*ψ*) is a factor of 10^2^ higher and a *S_ij_*_,*max*_ exists for 90% recovery up to an absolute pressure ratio of 10^3^. In addition, the *S_ij_*_,*max*_ is only around 10^2^ at the more reasonable *ψ* of 10^2^. Given this limitation, further increases in *S_ij_* from currently reported levels of CO_2_ separation membranes are unlikely to significantly enhance performance for very low feed fraction CO_2_ recovery.

Although not as severe as the *x_r_*_,*i*,0_ = 0.01 case, increasing the feed concentration to *x_r_*_,*i*,0_ = 0.10 still results in *S_ij_*_,*max*_ that may affect research targets for membrane performance. As shown in Figure 8c, *S_ij_*_,*max*_ exists for *R_i_* of 0.90 up to a *ψ* of 10^2^, with a *S_ij_*_,*max*_ of 10^2^ occurring at *ψ* = 45 and *S_ij_*_,*max*_ of 10^3^ at *ψ* = 80. An equally important result is the maximum purity, shown in Figure 8d. As recovery increases so do the gradients of the purity contour lines, showing that as *R_i_* is increased to 0.90 or higher the pressure ratio required to increase purity increases substantially. Thus high *R_i_* targets with low *x_r,i,_*_0_ feeds become increasingly limited in the maximum purity regardless of improvement in the selectivity of the membranes.

The dotted line denoted as *x_p_*_,*i*,*max*_ = 1 in each of the Figure 8 panels is the expected condition at which no asymptotic *S_ij_* occurs, meaning that the impurities can decline substantially even for *S_ij_* > 10^8^. This line was plotted using a relationship obtained by fitting

(34) $${\left.\psi_{i,\lim}\right|}_{x_{p,i,\max=1}}=x_{r,i,0}+\frac{1-x_{r,i,0}}{1-R_{i}}$$

where the pressure ratio that separates the purity-limited zone is defined as *ψ_i_*_,*lim*_, and is calculated as a function of the inlet mole fraction, *x_r_*_,*i*,0_, and target recovery, *R_i_*. Although empirically derived here, this equation fits the data accurately with the dotted lines straddling the gray region limits for *x_r_*_,*i*,0_ from 1% (Figure 8a) to 90% (Figure 8e). Although this equation provides no information on the specific limiting *S_ij_*, it clearly defines the region in which *S_ij_*_,*lim*_ exists for all conditions of a binary membrane separator. If a separator is operated at *ψ_i_*_,_ > *ψ_i_*_,*lim*_, then no maximum exists and an increase in *S_ij_* will always lead to a valuable increase in *x_p_*_,*i*_; however, if a separator is operated at *ψ_i_*_,_ < *ψ_i_*_,*lim*_, then a *x_p_*_,*i*,*max*_ is expected that results in a *S_ij_*_,*max*_ above which no further gains in purity can be expected.

For high *R_i_* targets coupled with low *x_r_*_,*i*,0_ feed sources, the *S_ij_*_,*max*_ limit could result in the limitation being near currently obtained research levels. Again taking CO_2_ capture as the example, if a 90% *R_i_* is coupled with a 10% *x_r_*_,*i*,0_ feed condition, then a *ψ_i_*_,*lim*_ of 9.1 is obtained. Yet, some studies have optimized membrane separators at high recovery and suggested *ψ_i_* of less than 10 [26]. More specifically, Kaldis et al. [30] reported that an increase in *S_ij_* from 50 to 90 did not increase the purity for a binary separator operating at *x_r_*_,*i*,0_ of 0.35 and *ψ_i_* of 7. Extracting information from the plot manually indicates the *R_i_* was around 97% for both *S_ij_*, which would result in a *ψ_i_*_,*lim*_ > 17 and a *S_ij_*_,*max*_ of 46 using the techniques outlined above. Thus, any *S_ij_* above 46 would not be expected to increase *x_p_*_,*i*_ in a simple 1D model and the results of the current study can be considered validated against similar simulation studies. However, it is unclear how the results would change if pressure drop, and diffusion/concentration polarization effects were considered in the model. Additionally, no experimental studies were found that observed limited changes with *S_ij_*, so the validity of this with real systems is not well understood.

## 4. Conclusions

A study on the maximum purity of a single-stage gas separator has been performed across a wide parameter space, with insight into the maximum effective selectivity of the membrane imposed by the purity limitations. Due to the tendency of non-idealities to reduce membrane separator performance, the simplified model is suggested to provide the maximum theoretical purity of a single-stage membrane separator. Note, however, that the conclusions found herein are not to be interpreted as the maximum potential purity of a multi-stage separator. For a more complex separator configuration with multiple stages and recycle loops, the results of this study should be applied individually to each stage using the separator feed condition after the recycle loop has been added. Furthermore, this study has focused on collecting a purified permeate stream, but another technique could potentially involve the removal of impurities and collecting a purified retentate stream. This study did not explore such options, but it is suggested that the removal of all impurities is not likely to be the economical choice if high purity is required because it would require that all components in a multi-component mixture be more permeable than the desired component. Nevertheless, this topic is left open to future study. A summary of the main observations and conclusions are:

1. An ideal membrane separator requires four input variables: the transport parameter, *θ_i_*, which balances the flow rate with the membrane permeance, the feed mole fraction, *x_r_*_,*i*,0_, the pressure ratio, *ψ_i_*, and the ideal selectivity, *S_ij_*;
2. For the majority of industrial applications, *x_r_*_,*i*,0_ is a fixed parameter based on the upstream processes or source. Similarly, to reduce waste and meet a target recovery (e.g., carbon capture requiring *R*_CO_2__ = 90%), *θ_i_* becomes fixed. Thus, optimization of the purity is accomplished primarily with *ψ_i_* and *S_ij_*;
3. *x_p,i_* increases with increasing *ψ_i_* until a maximum is reached at *ψ_i_* = ca. 10^2^. For *ψ_i_* > 10^2^, only *S_ij_* affects the purity. The required minimum *S_ij_*, or *S_ij_*_,*min*_, to achieve a target *x_p_*_,*i*_ can be obtained by using the analytical solution for *x_p_*_,*i*_ at *R_i_* = 0 and then dividing by a factor obtained from the plot of *S_ij_*/*S_ij_*_,*min*_ for *S_ij_* vs. *R_i_* (Figure 5b). If the *S_ij_*_,*min*_ is greater than the attainable technology, then the information can provide either new research objective targets or show that a multi-stage separator is the only option for the target purity;
4. Because *ψ_i_* > 10^2^ causes *ψ* to become very large for low *x_r_*_,*i*,0_, there exist regimes in which a maximum *S_ij_* is observed due to a maximum *x_p_*_,*i*_ limitation. To determine if the desired operating condition has a *S_ij_*_,*max*_ value, the target *R_i_* and *x_r_*_,*i*,0_ are input into Equation (33) to obtain *ψ_i_*_,*lim*_. If operating at *ψ_i_* < *ψ_i_*_,*lim*_ then a *S_ij_*_,*max*_ exists, but if operating with *ψ_i_* > *ψ_i_*_,*lim*_ then no maximum *S_ij_* exists. *S_ij_*_,*max*_ is dependent on *x_r_*_,*i*,0_, *ψ_i_*, and *R_i_*, so data must be gathered for the specific *x_r_*_,*i*,0_ of interest. Yet, as *x_r_*_,*i*,0_ decreases, the limitation with *S_ij_* becomes more important, potentially limiting any reason for further research on increasing *S_ij_*.

The above information is expected to be useful in setting membrane ideal selectivity targets for specified separations and membrane technologies.

## Figures and Tables

**Table 1:** Summary of required input variables for a shell-and-tube membrane system.

	Required Input	Variable	Unit
Geometry	Membrane length	*L*_m_	m
	Membrane radius	*r_m_*	m
	Shell radius	*r_s_*	m
Membrane	Permeation rate	*φ_i_*	mol m^−2^ s^−1^ Pa^−1^
	Ideal selectivity	*S_ij_*	-
Conditions	Feed rate	$\dot{V}$ *_f_*	m^3^ s^−1^
	Feed composition	*x_r_*_,*i*,0_	-
	Sweep rate	$\dot{V}$ *_s_*	m^3^ s^−1^
	Sweep composition	*x_p_*_,*i*,0_	-
	Reactor pressure	*P_r_*	Pa
	Permeate pressure	*P_p_*	Pa
	Temperature	*T*	K

**Table 2:** Summary of input variables for the membrane separator model.

	Required Input	Variable	Unit
Dimensionless Parameters	Ideal selectivity	*S_ij_*	-
	Feed composition	*x_r_*_,*i*,0_	-
	Transport parameter	*θ_i_*	-
	Pressure ratio	*ψ*	-

**Table 3:** Minimum required membrane selectivity to obtain target *x_p,i_* and *R_i_* for ideal CO_2_ separation (e.g., CO_2_/N_2_ or CO_2_/CH_4_) with 10% feed concentration. Calculated using *ψ_i_* = 10^2^.

Target Purity (*x_p,i_*)	Required Selectivity (*S_ij_*)		
	*R_i_* = 0%	*R_i_* = 90%	*R_i_* = 95%
70%	21	57	72
90%	89	240	300
99%	950	2600	3200
99.9%	5000	14,000	17,000

## Data Availability

The original contributions presented in the study are included in the article/Supplementary Materials, further inquiries can be directed to the corresponding author.

## Supplementary Materials

The following supporting information can be downloaded at https://www.mdpi.com/article/10.3390/membranes14060143/s1, Figure S1: Product recovery, *R_i_* (a,c,e,g), and permeate purity, *x_p_*_,*i*_ (b,d,f,h), dependence on the transport parameter, *θ_i_*, and modified dimensionless pressure ratio *ψ_i_*. (a,b) *x_r_*_,*i*,0_ = 0.10, *S_ij_* = 10, (c,d) *x_r_*_,*i*,0_ = 0.7, *S_ij_* = 10, (e,f) *x_r,i,0_* = 0.10, *S_ij_* = 500, (g,h) *x_r_*_,*i*,0_ = 0.7, *S_ij_* = 500.

## Nomenclature

*A_m_*	membrane permeable area
*A_p_*	cross-sectional area of the permeate
*A_r_*	cross-sectional area of the retentate
*c_p,i_*	bulk concentration of species *i* in the permeate
*c_r,i_*	bulk concentration of species *i* in the retentate
*J_i_*	flux of species *i* through the membrane
*L_m_*	membrane length
*ṅ*_*r,i,*0_	retentate inlet molar flow rate of species *i*
*ṅ*_*r,i,*1_	retentate outlet molar flow rate of species *i*
*ṅ_p_*	permeate outlet total molar flow rate
*ṅ_p,i_*	permeate outlet molar flow rate of species *i*
*P_m_*	membrane circumference
*P_p_*	permeate total pressure
*P_r_*	retentate total pressure
*P*_0_	standard pressure (100 kPa)
*R*	universal gas constant (8.314 J mol^−1^ K^−1^)
*R_i_*	recovery of species *i*
*r_m_*	membrane radius
*r_s_*	shell radius
*S_ij_*	ideal selectivity of component *i* vs. *j*
*S_ij,max_*	maximum selectivity defined as selectivity to obtain 99% of maximum purity
*S_ij,min_*	minimum selectivity for specified purity obtained from solution at zero recovery
*T*	feed temperature
*T*_0_	standard temperature (273.15 K)
*u_r,z_*	retentate axial fluid velocity
*u*_*r,z,*0_	feed inlet axial fluid velocity
*u_p,z_*	permeate axial fluid velocity
*u*_*p,z,*0_	sweep inlet axial fluid velocity
$\dot{V}$ *_f_*	feed volumetric flow rate
$\dot{V}$ *_s_*	sweep volumetric flow rate
*x_r,i_*	retentate mole fraction of species *i*
*x*_*r,i,*0_	feed inlet mole fraction of species *i*
*x_p,i_*	permeate mole fraction of species *i* (purity of species *i* at outlet)
*x*_*p,i,*0_	sweep inlet mole fraction of species *i*
*x_p,i,max_*	maximum potential purity obtained from analytical solution at zero recovery
*z*	axial position
*ξ_p,i_*	impurity fraction in permeate of component *i*
*ξ_p,i,error_*	impurity fraction error between two specified conditions
*φ_i_*	permeation constant of component *i*
*θ_i_*	dimensionless transport parameter (modified Péclet number)
*θ_i,offset_*	fractional change in the difference of *θ_i_* between two specified conditions
*ζ*	dimensionless axial position along the membrane
*ν_r,z_*	dimensionless retentate flow velocity
*ν_p,z_*	dimensionless permeate flow velocity
*ψ*	reduced pressure ratio
*ψ_i_*	modified reduced pressure ratio of component *i*
*ψ_i,lim_*	limiting modified reduced pressure ratio of component *i*
