# Supplementary material for: On the Maximum Obtainable Purity and Resultant Maximum Useful Membrane Selectivity of a Membrane Separator
Source: Membranes (Basel). 2024 Jun 19;14(6):143. doi: 10.3390/membranes14060143 (PMC11206096; doi:10.3390/membranes14060143)
Supplement: Supplementary file 1 [file membranes-14-00143-s001.zip › membranes-3042371-supplementary.pdf]

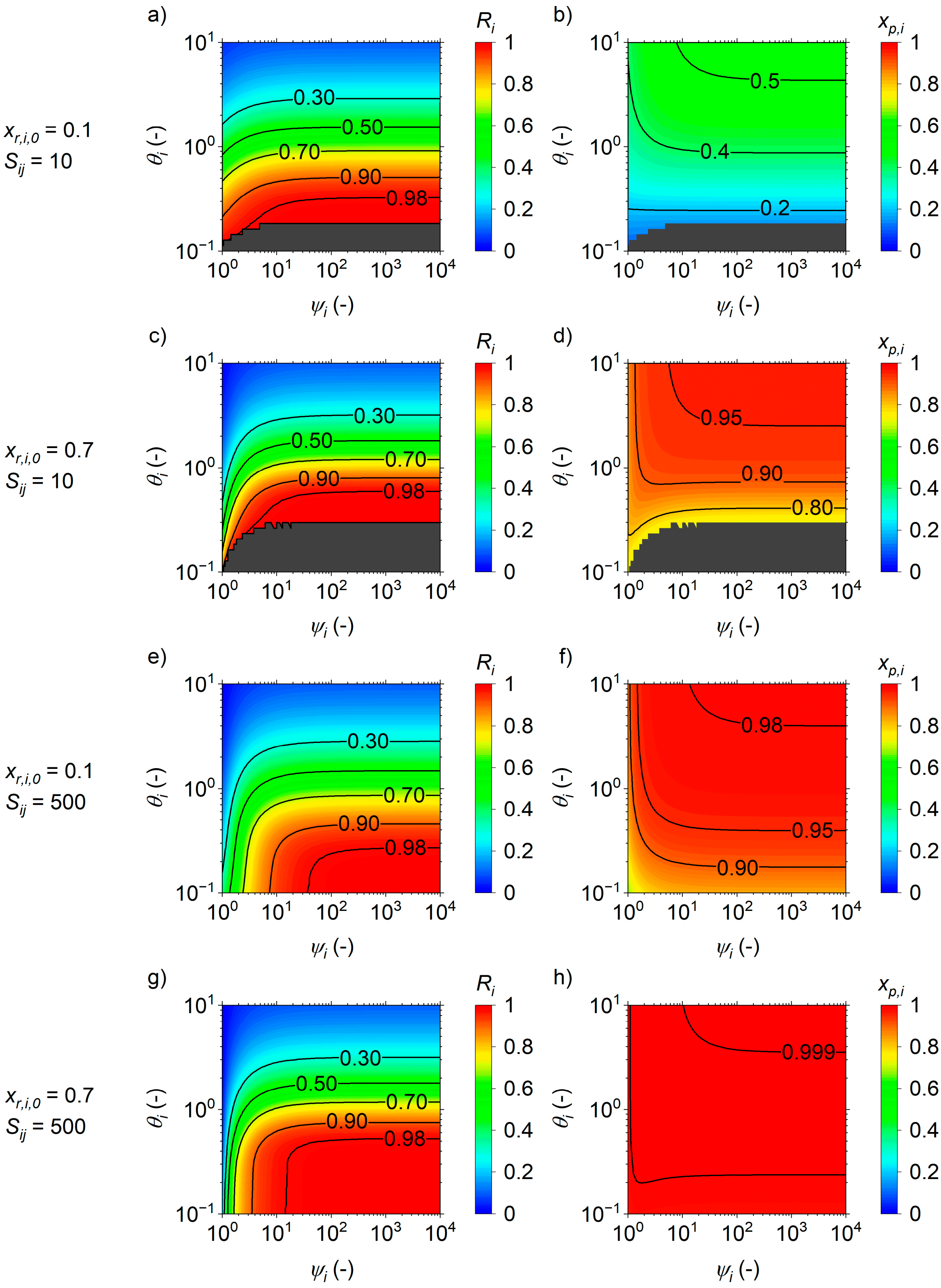

Figure S1. Product recovery, R_i (**a,c,e,g**), and permeate purity, $x_{p,i}$ (**b,d,f,h**), dependence on the transport parameter, θ_i, and modified dimensionless pressure ratio ψ_i. (**a,b**) $x_{r,i,0} = 0.1$, $S_{ij} = 10$, (**c,d**) $x_{r,i,0} = 0.7$, $S_{ij} = 10$, (**e,f**) $x_{r,i,0} = 0.1$, $S_{ij} = 500$, (**g,h**) $x_{r,i,0} = 0.7$, $S_{ij} = 500$.
